# Changes in carotid artery texture by ultrasound and elastin features in a murine model

**DOI:** 10.3389/fcvm.2023.1215449

**Published:** 2023-07-25

**Authors:** Carol Mitchell, Rashid Al Mukaddim, Yuming Liu, Melissa Graham, Jens C. Eickhoff, Ashley M. Weichmann, Matthew C. Tattersall, Claudia E. Korcarz, James H. Stein, Tomy Varghese, Kevin W. Eliceiri

**Affiliations:** ^1^ Department of Medicine, University of Wisconsin-Madison, Madison, WI, United States; ^2^ Department of Medical Physics, University of Wisconsin-Madison, Madison, WI, United States; ^3^ Center for Quantitative Cell Imaging, University of Wisconsin-Madison, Madison, WI, United States; ^4^ Comparative Pathology Laboratory, Research Animal Resources and Compliance, University of Wisconsin-Madison, Madison, WI, United States; ^5^ Department of Biostatistics and Medical Informatics, University of Wisconsin-Madison, Madison, WI, United States; ^6^ Carbone Cancer Center, Small Animal Imaging and Radiotherapy Facility, University of Wisconsin-Madison, Madison, WI, United States; ^7^ Department of Biomedical Engineering, University of Wisconsin-Madison, Madison, WI, United States; ^8^ Morgridge Institute for Research, Madison, WI, United States

**Keywords:** atherosclerosis, pre-clinical murine model, carotid, ultrasound imaging, texture features, elastin quantification

## Abstract

**Objective:**

In humans, arterial grayscale ultrasound texture features independently predict adverse cardiovascular disease (CVD) events and change with medical interventions. We performed this study to examine how grayscale ultrasound texture features and elastin fibers change in plaque-free segments of the arterial wall in a murine model prone to atherosclerosis.

**Methods:**

A total of 10 Apoe^tm1Unc/J^ mice (*n* = 5 male, *n* = 5 female) were imaged at 6, 16, and 24 weeks of age. Two mice were euthanized at 6 and 16 weeks and the remaining mice at 24 weeks. Texture features were extracted from the ultrasound images of the distal 1.0 mm of the common carotid artery wall, and elastin measures were extracted from histology images. Two-way analysis of variance was used to evaluate associations between week, sex, and grayscale texture features. Texture feature and elastin number comparisons between weeks were conducted using the sex-by-week two-way interaction contrasts. Sex-specific correlations between the number of elastin fibers and grayscale texture features were analyzed by conducting non-parametric Spearman's rank correlation analyses.

**Results:**

Arterial wall homogeneity changed significantly in male mice from 6 to 24 weeks, with a mean (SD) of 0.14 (0.03) units at 6 weeks and 0.18 (0.03) units at 24 weeks (*p* = 0.026). Spatial gray level dependence matrices-homogeneity (SGLD-HOM) also correlated with carotid artery plaque score (*r_s_* = 0.707, *p* = 0.033). Elastin fibers in the region of interest decreased from 6 to 24 weeks for both male and female mice, although only significantly in male mice. The mean (SD) number of elastin fibers for male mice was 5.32 (1.50) at 6 weeks and 3.59 (0.38) at 24 weeks (*p* = 0.023). For female mice, the mean (SD) number of elastin fibers was 3.98 (0.38) at 6 weeks and 3.46 (0.19) at 24 weeks (*p* = 0.051).

**Conclusion:**

Grayscale ultrasound texture features that are associated with increased risk for CVD events in humans were used in a murine model, and the grayscale texture feature SGLD-HOM was shown to change in male mice from 6 weeks to 24 weeks. Structural alterations of the arterial wall (change in elastin fiber number) were observed during this time and may differ by sex.

## 1. Introduction

In humans, arterial grayscale texture features independently predict adverse cardiovascular disease (CVD) events and change with medical interventions (1–3). Although arterial wall echogenicity [using the texture feature grayscale median (GSM)] has been used to examine relationships of echogenicity/echolucency to CVD risk factors and events, other grayscale arterial texture features (i.e., homogeneity and contrast) are new. These new texture features are being used increasingly as research outcomes; however, the anatomical findings they represent in plaque-free segments of the carotid arterial wall remain unknown.

Some investigators have postulated that arteries that are more echolucent (dark gray) are indicative of infiltration of lipids and inflammatory cells based on associations between ultrasound characteristics of carotid plaques and endarterectomy specimens (1, 3–17). For example, Lal et al. (12) demonstrated that grayscale values could indeed be used to predict the histologic content of carotid plaque from endarterectomy specimens and suggested that grayscale characteristics of carotid plaques may be a useful tool to help with risk stratification for identifying plaques that are at risk for rupture. However, in the Multi-Ethnic Study of Atherosclerosis (18), grayscale changes in plaques did not predict future atherosclerotic CVD events, so we and other investigators extracted texture features from plaque-free segments of the common carotid artery (CCA) wall and demonstrated that grayscale texture features and measures of echolucency are associated with several traditional and inflammatory CVD risk factors (e.g., hypertension, body mass index, diabetes mellitus, interleukin-6, and fibrinogen) and predict adverse CVD events (1, 6, 7, 19).

Although these early studies demonstrated relationships between arterial texture, CVD risk factors, and adverse CVD events, their actual relationship to arterial wall microstructure and cellular composition in plaque-free segments have not been well studied. If these texture features are related to arterial wall microstructure and cellular composition due to early injury (i.e., prior to atherosclerotic plaque formation), texture features have the potential to become a powerful research tool for studying early arterial injury and remodeling associated with arterial disease and treatment of risk factors. Ultrasound is safe, widely available, and inexpensive compared to other imaging modalities; thus, it is an ideal tool for monitoring arterial health across the life span (1, 8).

Subsequently, we and other investigators extracted texture features from plaque-free segments of the common carotid artery wall and demonstrated that grayscale texture features and measures of echolucency are associated with several traditional and inflammatory CVD risk factors (e.g., hypertension, body mass index, diabetes mellitus, interleukin-6, and fibrinogen) and predict incident adverse CVD events (1, 6, 7, 19–21). Nevertheless, the relationships of these novel measures to arterial wall microstructure and cellular composition in plaque-free segments have not been studied previously. If these texture features are related to arterial wall microstructure and cellular composition with early injury (i.e., prior to atherosclerotic plaque formation), texture features have the potential to become a powerful research tool for studying early arterial injury and remodeling associated with arterial disease and treatment of risk factors.

Our objective was to examine how novel uses of ultrasound grayscale texture features are associated with an arterial structure based on the presence and quantity of elastin fibers. We hypothesized that with age-related elastin fragmentation and arterial injury due to atherogenesis, arterial walls will demonstrate fewer shades of gray resulting in a more homogenous phenotype with lower contrast and higher homogeneity, a phenotype that has been associated with an increased risk for adverse CVD events in humans (1).

## 2. Materials and methods

### 2.1. Mice models

**Figure 1:**
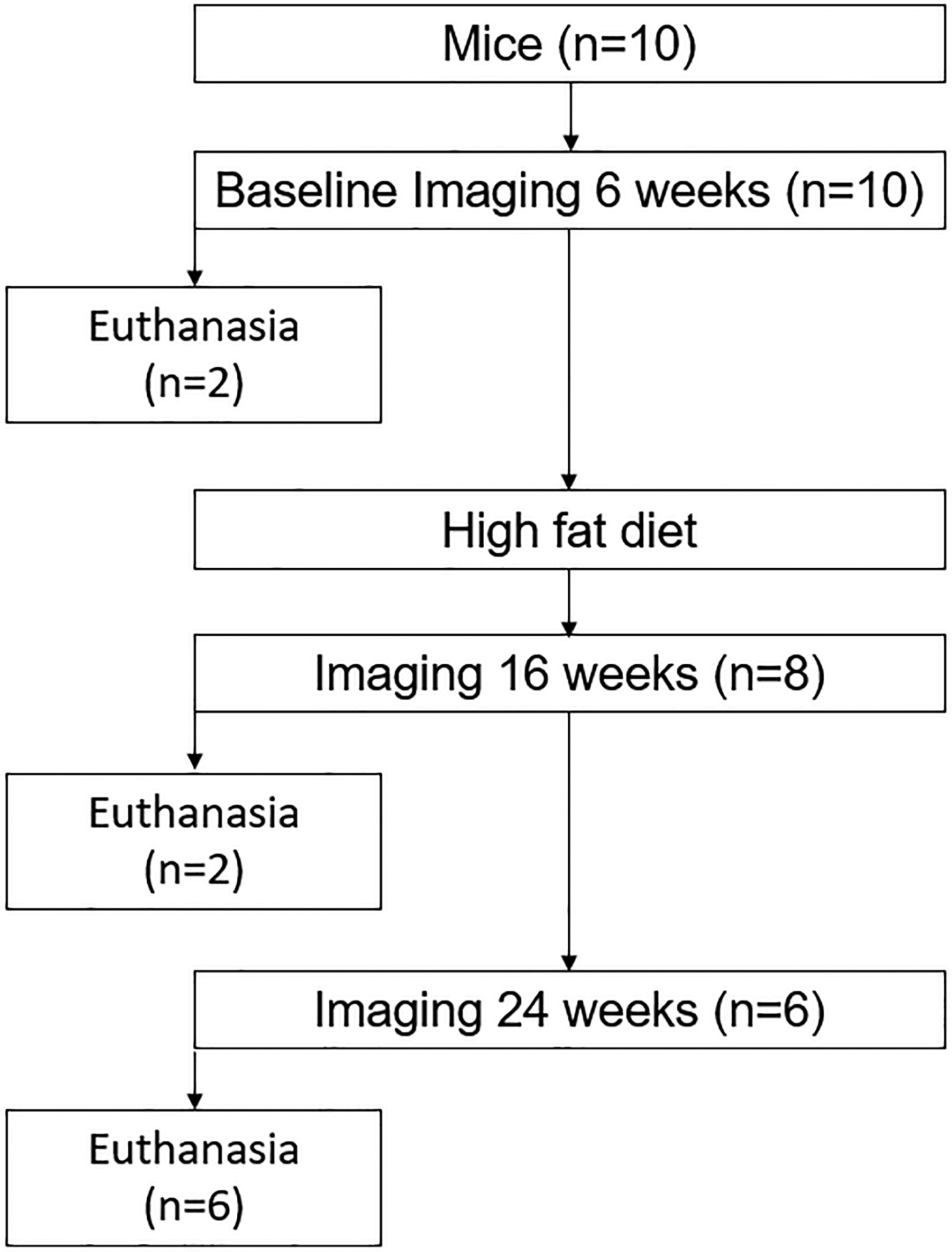
Flowchart of the longitudinal study to evaluate the associations between ultrasound grayscale texture features and arterial wall structure. A total of 10 Apoe^tm1Unc/J^ mice (*n* = 5 male, *n* = 5 female) were imaged at 6, 16, and 24 weeks of age. Euthanasia was performed for two mice at 6 weeks (one male, one female), two mice at 16 weeks (one male, one female), and six mice at 24 weeks (three male, three female) to evaluate the temporal variation of structural change.

Ten Apoe^tm1Unc/J^ mice (*n* = 5 male, *n* = 5 female, B6.129P2 Apoe^tm1Unc/J^, The Jackson Laboratory, Bar Harbor, ME, United States) were imaged at 6, 16, and 24 weeks of age. After baseline imaging, all mice were fed a high-fat diet [Envigo TD.88137, adjusted calorie diet (42% from fat), East Millstone, NJ, United States] *ad libitum* until 24 weeks. Euthanasia was performed via CO_2_ inhalation for two mice at 6 weeks (one male, one female), two mice at 16 weeks (one male, one female), and six mice at 24 weeks (three male, three female) (Figure 1). Tissue was harvested from all mice including the superior aortic arch, bilateral common carotid arteries, and internal and external carotid arteries. This study examined changes in the carotid artery wall associated with atherogenesis and included only ApoE mice, which are prone to atherosclerosis (22). It was approved by the University of Wisconsin School of Medicine and Public Health, Institutional Animal Care and Use Committee. All experiments were conducted in accordance with the relevant guidelines and regulations associated with this protocol.

### 2.2. High-frequency ultrasound imaging

**Figure 2:**
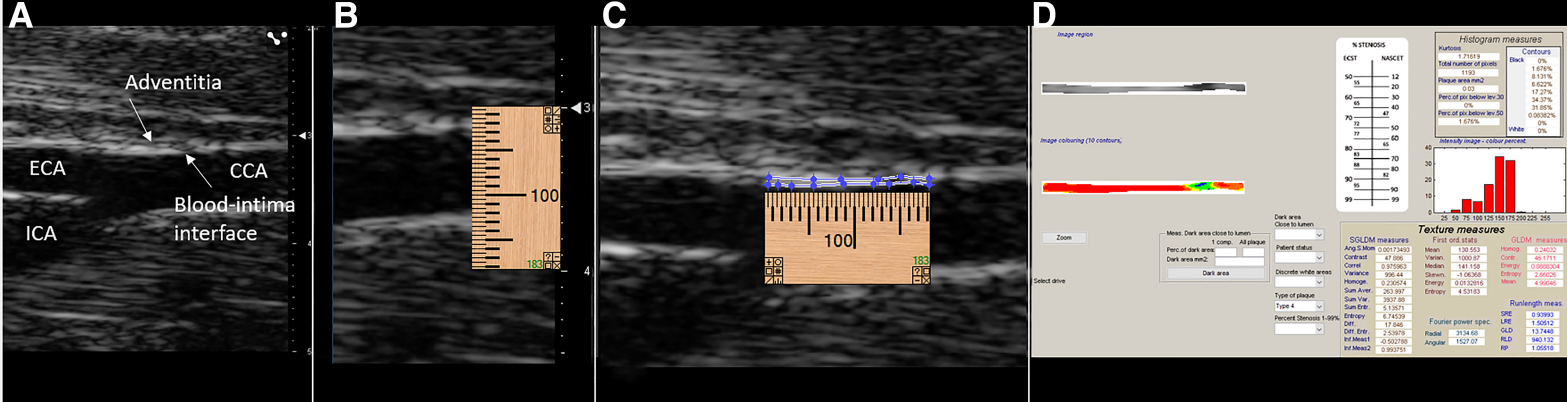
Image segmentation process. (**A**) RAW bitmap image exported from the VisualSonics workstation. (**B**) Placement of an online ruler tool to mark 1.00 mm length when the image is zoomed one time in the LifeQ Medical software (LifeQ Medical Ltd, Nicosia, Cyprus). (**C**) Manual segmentation of the intima–media for a distance of 1.00 mm of the near wall of the distal common carotid artery. (**D**) Software output of texture features with grayscale and colorized segmentation based on grayscale value (color grayscale values: black = 0–25, blue = 26–50, green 51–75, yellow = 76–100, orange 101–125, red = 126–255).

*In vivo* image acquisition methods have been described previously (23). Briefly, ultrasound imaging was performed with the Vevo 2100 high-frequency ultrasound system (Fujifilm VisualSonics, Toronto, ON, Canada) and an MS 700 linear array transducer (center frequency 50 MHz) at 1,000 frames per sec in ECG-gated kilohertz visualization (EKV) mode. All imaging was performed with simultaneous acquisition of ECG and respiratory signals. The common carotid arteries were imaged in the longitudinal plane from an anterior approach demonstrating the external carotid artery (ECA) in continuation with the common carotid artery. The internal carotid artery (ICA) was also depicted in this image (Figure 2). The image plane was optimized to demonstrate the blood intima interface and media adventitia interface on the near wall. EKV mode was used to acquire all B-mode grayscale images used for texture feature extraction. EKV mode requires high frame rate data collection to extract a single cardiac cycle of processed B-mode data based on ECG and respiration signal gating. Therefore, the frame rate was set to 1,000 FPS. However, the grayscale analysis was only limited to a single frame of data at the end-diastole of a cardiac cycle, which was selected based on visual inspection of the EKV cine loop and the R wave [first upward deflection on the ECG after the *p* wave; the *p* wave is the small upward deflection on the ECG that represents atrial depolarization (24)] on ECG. The use of EKV mode ensured that consistent post-acquisition filtering was applied to all images used for grayscale analyses (Table 1, EKV processing mode). The single frame was exported as a bitmap image for offline analyses. A preset was used for all imaging sessions, and parameters are defined in Table 1.

**Table 1:** Imaging preset parameters.

Parameter	Value
Depth	5 mm
Depth offset	2 mm
Width	2.73 mm
Dynamic range	60 dB
Line density	High
Frame rate in EKV mode	1,000 Hz
EKV processing mode	Presentation
Focus	3 mm
Overall gain	34.98 dB
Time gain compensation	Midline (standardized)

### 2.3. Grayscale texture feature extraction

Grayscale texture features of the distal 1.0 mm of the near wall of the common carotid arteries were extracted using LifeQ Medical Carotid Plaque Analysis Software (LifeQ Medical Ltd, Nicosia, Cyprus) (1, 8–10, 25–30). All images were normalized so that the darkest pixel in the blood was assigned a grayscale value of 0 and the middle two-fourths of a plaque-free segment of the adventitia wall were assigned a grayscale value of 190 (1, 8–10, 25–27, 29–31). All images were at a pixel density of 190 pixels/mm. Grayscale texture features extracted for this study were grayscale median value (GSM), entropy, contrast [using the gray level difference statistic method (GLDM)], angular second moment (ASM), and inverse difference moment homogeneity (HOM) [using the spatial gray-level dependence matrices method (SGLD)] (1, 2, 8, 29, 30). These features were chosen based on findings in human studies (1, 8, 29). The normalized image was zoomed in once using the reading software. Manual segmentation was performed on the normalized image by using an online ruler tool (Microsoft Windows Ruler) to identify the length of 1.00 mm. The ruler length was then placed below the blood intima interface of the near wall of the CCA, and the intima–media (IM) was manually traced (Figure 2).

### 2.4. Tissue collection and histology

The abdomen and thorax were opened through a ventral midline incision, and the ventral chest plate was removed. The thymus and lungs were removed to allow for visualization of the aortic arch and branching vessels. Grossly identifiable plaques were described and measured. After exposing the branch point segments (internal, external, and rostral-most few millimeters of the common carotid arteries) bilaterally, the branch point segments adhered to a small piece of tissue wrap (BioSavers®, Innovative Pathology Concepts, Inc., Baltimore, MD, United States) in anatomic orientation. Small amounts of differently colored tissue ink (Davidson Marking System®, Bradley Products Inc., Bloomington, MN, United States) were applied to the exposed internal and external carotid arteries to aid in the orientation of the tissue specimens once the arteries were removed. The internal, external, and common carotid arteries were each transected, and the branch point segments were removed in anatomic orientation and adhered to the tissue wrap. Each branch point segment was sandwiched with another piece of tissue wrap between two biopsy foam pads (Simport, Beloeil, QC, Canada) and placed in a tissue cassette. The remaining portions of the common carotid arteries, in addition to the proximal aortic arch, were removed *en bloc* from the thoracic cavity and placed in the cassette in a similar manner, with the rostral ends of the transected common carotid arteries identified with small amounts of tissue ink. The tissue cassette was then placed in 10% neutral buffered formalin until fixed. The cassette then underwent standard paraffin processing in an automatic tissue processor. The tissues were embedded as flat as possible and as close to the anatomic imaging position as possible within a paraffin block by the submitting pathologist. The branch point segments were sectioned entirely through the regions of interest in continuous 5 μm sections, with each section captured sequentially on serial slides. All slides were stained with hematoxylin and eosin (H&E). Cytoseal™ 60 mounting medium (Thermo Fisher Scientific, Waltham, MA, United States) was used with #1.5 coverslips. A pathologist reviewed all H&E-stained histologic sections, evaluating the branch point segments for plaque presence in the region of interest (ROI) and nearby. The ROI was defined and identified as the approximately 1 mm region on the near (i.e., anterior/ventral) wall of the CCA wall as it extends toward the external carotid artery (identified by tissue ink). Sections were evaluated for the presence of foam cells, cholesterol clefts, spindle cells, eosinophilic material, media expansion, and any other abnormalities. All H&E slides including the ROIs were digitally scanned (see below). The central slide(s) for each mouse which demonstrated the best view of the ROIs were selected for additional combined staining with Masson's trichrome and Verhoeff's Van Gieson. After examining all sampled levels of each carotid segment, 17 slides were selected by the pathologist to include the level best representing each ROI (i.e., including as much of the ROI as possible with minimal artifact) for H&E destaining followed by re-staining to identify collagen (Masson's trichrome) and elastin (Verhoeff's Van Gieson). Selecting in this way ensured the best possible match to the ultrasound plane ROI (distal 1.0 mm of the near wall of the CCA as it extended to the external carotid artery). In some cases, a mouse's left and right ROIs were both sampled well at the same level, such that only one rather than two slides per mouse were needed. The pathologist identified the distal 1.0 mm of the near wall of the CCA as it branched into the external carotid artery and digitally segmented the intima–media and adventitia layers of the common carotid artery wall for microscopic imaging. Combination-stained Masson's trichrome and Verhoeff's Van Gieson slides were also examined by the pathologist, who then assisted in annotating elastin fibers for analysis of fiber separation. A bilateral plaque score was calculated for each mouse (0–8) with one point awarded for plaque presence in each carotid arterial segment (e.g., common, bulb, internal, and external; right and left).

### 2.5. Plaque score

At the time of gross pathology examination, the pathologist reported plaque presence and location. Four arterial segments on both the right and left sides (CCA, carotid bulb, internal carotid artery, and external carotid artery) were used to determine the plaque score. One point was awarded for plaque presence in each arterial segment. Thus, plaque scores could range from 0 to 8.

### 2.6. Elastin quantification

H&E slides were scanned at 20× using an Aperio CS2 Digital Pathology Scanner (Leica Biosystems, Buffalo Grove, IL, United States) by the Translational Research Initiatives in Pathology (TRIP) Laboratory at the University of Wisconsin-Madison. A pathologist annotated the ROIs including the adventitia and intima–media layers on the Aperio images. A traditional bright-field light microscope (BX53; Olympus Corp.) with Olympus UPlanFL 40×/NA = 0.75 objective lenses (Olympus Corp.) was used to image the corresponding ROIs on the combination-stained Masson's trichrome and Verhoeff's slides to provide higher resolution bright-field images of the elastin fibers. Based on the manual annotations of the elastin fibers performed in collaboration by the imaging scientist and pathologist, a Fiji (32) plugin “Weka Segmentation” (33) was used to highlight elastin fibers in each ROI, and a semiautomatic MATLAB (Natick, MA, United States: The MathWorks Inc.) script was then developed to quantify the number and spacing of the representative elastin fibers in each ROI for both the right and left common carotid arteries. Specifically, bright-field elastin ROI images were first manually labeled to identify 1–6 lines roughly perpendicular to the vessel wall by referring to the images with highlighted elastin fibers. The number of lines in each image was dependent on the workable ROI length (excluding artifacts) but was adjusted to include the representative elastin fiber patterns and the representative thickness of the IM layer. Along each annotated line, the two edge points of intima–media layer as well as the points associated with elastin positions within the intima–media layer were marked using the MATLAB script to calculate the thickness of the intima–media layers, the number of elastin fibers, the distances between adjacent elastin points, and the summary statistics of the elastin distances (minimum, maximum, mean, median, and standard deviation). The thickness of the intima–media layers, maximum distance between elastin fibers, median distance between elastin fibers, minimum distance between elastin fibers, and number of elastin fibers were measured in the distal 1.0 mm region of interest from histology slide digital images.

Methods for identifying the ROI were reviewed by a single pathologist, imaging scientist, and ultrasound reader. The methods described above were employed to standardize and best match the ROI from the *in vivo* ultrasound image to the histopathology image.

### 2.7. Statistical analysis

Grayscale texture features [GSM, gray level difference statistic method—contrast (GLDM-CON), SGLD-ASM, and SGLD-HOM] were summarized by means and standard deviation, stratified by sex and week. A two-way analysis of variance (ANOVA) was used to evaluate associations between week, sex, and grayscale texture features. Comparisons of grayscale features between weeks were conducted using the sex-by-week two-way interaction contrasts. The comparisons of the number of elastin fibers between weeks were conducted using the same analytic approach. Residual plots were examined to verify the model assumptions. The results were presented in graphical format using box and whisker plots. The sex-specific correlations between the number of elastin fibers and grayscale texture features and plaque score were analyzed by conducting non-parametric Spearman's rank correlations analyses after splitting the data file by sex and using both the left- and right-side measurements. All the reported *p*-values are two-sided, and *p* < 0.05 was used to define statistical significance. Statistical analysis was conducted using SAS software (SAS Institute, Cary, NC, United States), and version 9.4. Origin (Version 2020, OriginLab Corporation, Northampton, MA, United States) was used for box and whisker plots.

## 3. Results

### 3.1. Plaque presence

The gross examination of the two mice euthanized at 6 weeks demonstrated no atherosclerotic plaques in the common carotid, carotid bulb, and internal or external carotid artery segments. At 16 weeks, the gross examination of the same regions demonstrated no atherosclerotic plaque in the female mouse and three atherosclerotic plaques in the carotid arterial system of the male mouse. At 24 weeks, six mice were euthanized (*n* = 3 male, *n* = 3 female). All six mice had atherosclerotic plaques in their carotid arteries. The range of plaque scores for male and female mice at 6, 16, and 24 weeks is shown in Table 2.

**Table 2:** Plaque scores for each mouse at 6, 16, and 24 weeks.

Mouse ID	Sex	Week	Left-side plaque score	Right-side plaque score
5	Male	6	0	0
2	Female	6	0	0
4	Male	16	1	2
4	Female	16	0	0
1	Male	24	2	2
2	Male	24	2	2
3	Male	24	2	2
1	Female	24	4	2
3	Female	24	2	3
5	Female	24	4	1

Plaque score derived by assigning 1 point for plaque in each of the following arterial segments (CCA, bulb, ICA, ECA).

### 3.2. Grayscale texture changes from 6 weeks to 16 weeks and 6 weeks to 24 weeks

#### 3.2.1. Grayscale median (GSM)

**Figure 3:**
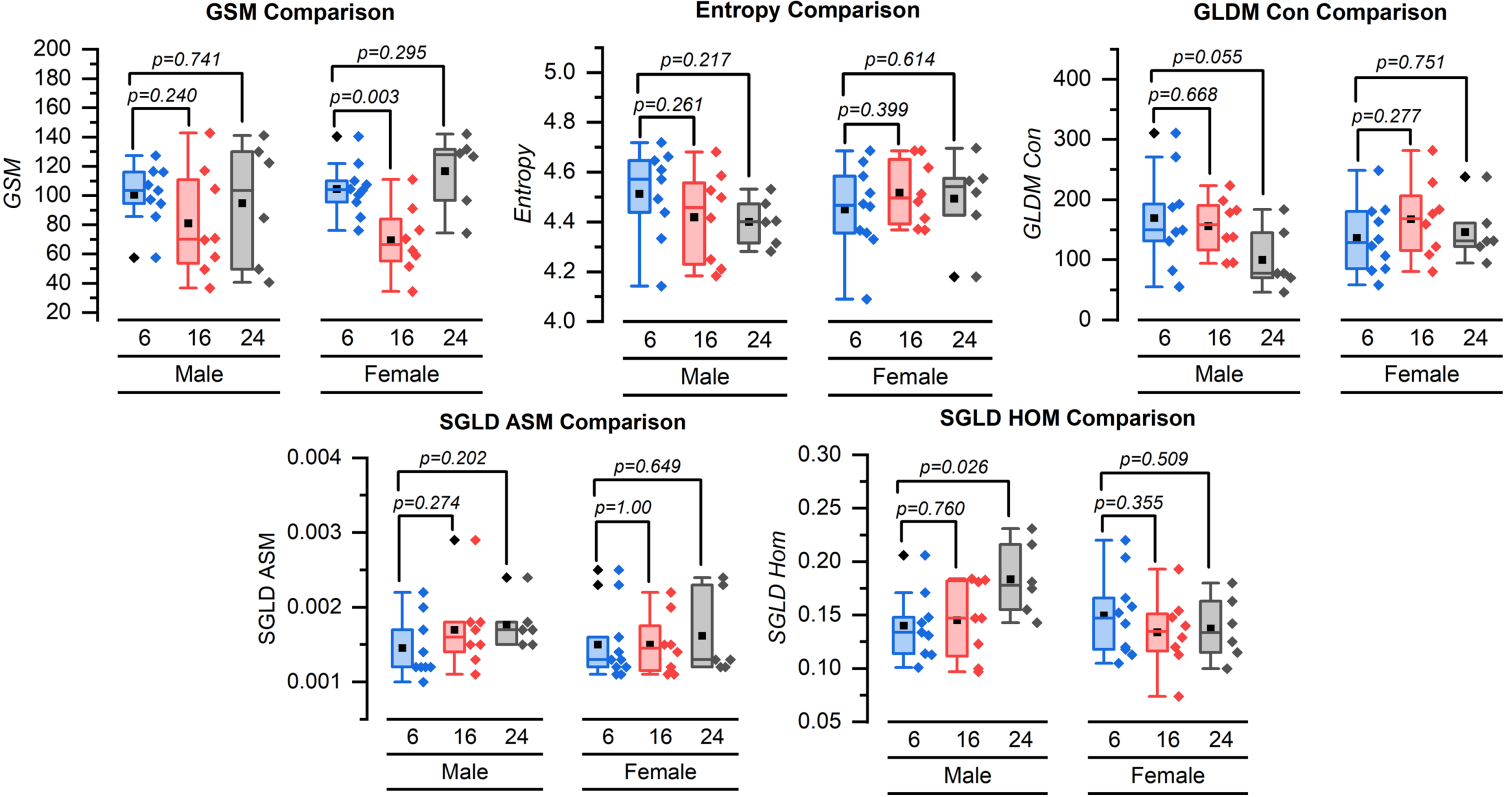
Box and whisker plots for the grayscale texture features for 6, 16, and 24 weeks. GSM = grayscale median value, GLDM-CON = gray level difference statistic method—contrast, SGLD-ASM = spatial gray level dependence matrices method angular second moment, and SGLD-HOM = spatial gray-level dependence matrices method homogeneity. Symbol definitions: ▪ = mean, ♦ = outlier.

For female mice, the mean [standard deviation (SD)] GSM significantly decreased from 104.57 (17.99) units to 69.59 (23.76) units at 16 weeks (*p* = 0.003). However, the change from 104.57 (17.99) units at 6 weeks to 116.79 (25.75) units at 24 weeks was not significant (*p* = 0.295). In male mice, the GSM did not change significantly over time. The mean GSM values at 6, 16, and 24 weeks were 100.67 (20.62) units, 81.15 (36.62) units (*p* = 0.240), and 94.80 (42.92) units (*p* = 0.741), respectively (Figure 3).

#### 3.2.2. Grayscale entropy

Neither females nor males demonstrated a significant change in entropy from 6 weeks of imaging to 16 or 24 weeks. The mean (SD) grayscale entropy value for the females was 4.45 (0.17) units at 6 weeks, 4.52 (0.13) units at 16 weeks (*p* = 0.399), and 4.49 (0.18) units at 24 weeks (*p* = 0.614). The mean entropy values for males were 4.51 (0.18) units at 6 weeks, 4.42 (0.19) units at 16 weeks (*p* = 0.261), and 4.40 (0.09) units at 24 weeks (*p* = 0.217) (Figure 3).

#### 3.2.3. Gray-level difference statistic method (GLDM)—contrast (CON)

The mean GLDM-CON values for female mice at 6, 16, and 24 weeks were 136.50 (57.91), 167.71 (65.93), and 146.28 (49.78) units with no statistically significant difference (*p* = 0.277 and *p* = 0.751 for 6–16 weeks and 6–24 weeks, respectively). For male mice, the mean GLDS-CON value was 169.61 (82.36) units at 6 weeks, 155.93 (47.54) units at 16 weeks (*p* = 0.668), and lower at 100.01 (52.61) units at 24 weeks (*p* = 0.055) (Figure 3).

#### 3.2.4. Spatial gray-level dependence matrix method (SGLD)-angular second moment (ASM)

The SGLD-ASM values did not change significantly for female or male mice from 6 to 16 weeks or 6 to 24 weeks (Figure 3). The mean SGLD-ASM value for female mice was 0.0015 (0.0005) units at 6 weeks, 0.0015 (0.0004) units at 16 weeks, and 0.0016 (0.0006) units at 24 weeks (*p* = 1.000, for 6–16 weeks and *p* = 0.649 for 6–24 weeks). The mean SGLD-ASM value for male mice was 0.0015 (0.0004) units at 6 weeks, 0.0017 (0.0005) units at 16 weeks, and 0.0018 (0.0003) units at 24 weeks (*p* = 0.274 and *p* = 0.202 for 6–16 and 6–24 weeks, respectively) (Figure 3).

#### 3.2.5. SGLD-inverse difference moment homogeneity (HOM)

The SGLD-HOM values did not change significantly for female mice from 6 to 16 or 6 to 24 weeks. SGLD-HOM was 0.15 (0.04) units at 6 weeks, 0.13 (0.03) units at 16 weeks, and 0.14 (0.03) units at 24 weeks (*p* = 0.355 and *p* = 0.509 for 6–16 and 6–24 weeks, respectively). For male mice, the mean value for SGLD-HOM was 0.14 (0.03) units at 6 weeks and 0.15 (0.04) units at 16 weeks (*p* = 0.760 for 6–16 weeks) and significantly increased to 0.18 (0.03) units at 24 weeks (*p* = 0.026 for 6–24 weeks) (Figure 3).

### 3.3. Elastin fibers

**Figure 4:**
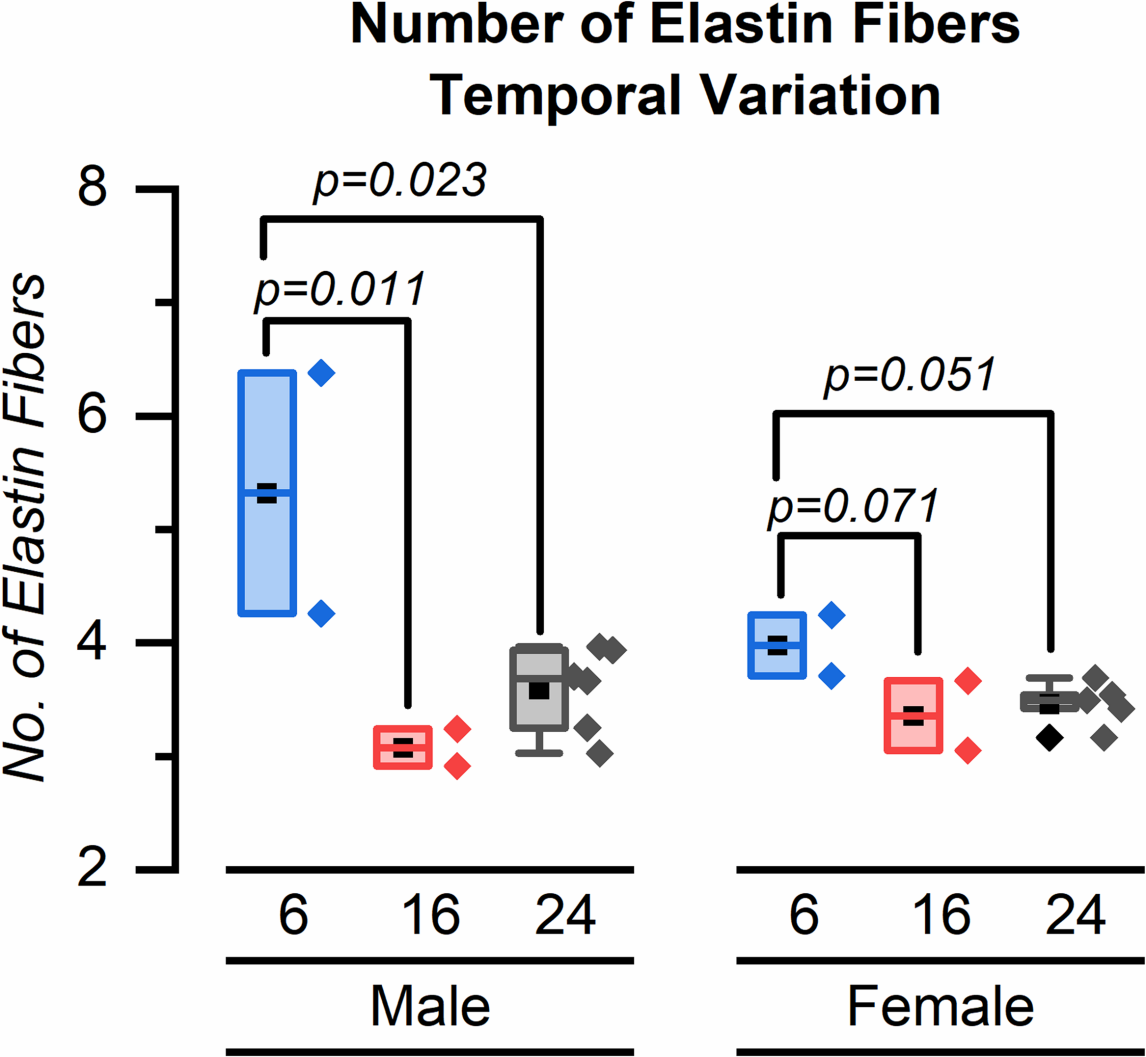
Distribution of elastin fibers for male and female mice at 6, 16, and 24 weeks. Symbol definitions: ▪ = mean, ♦ = outlier.

**Figure 5:**
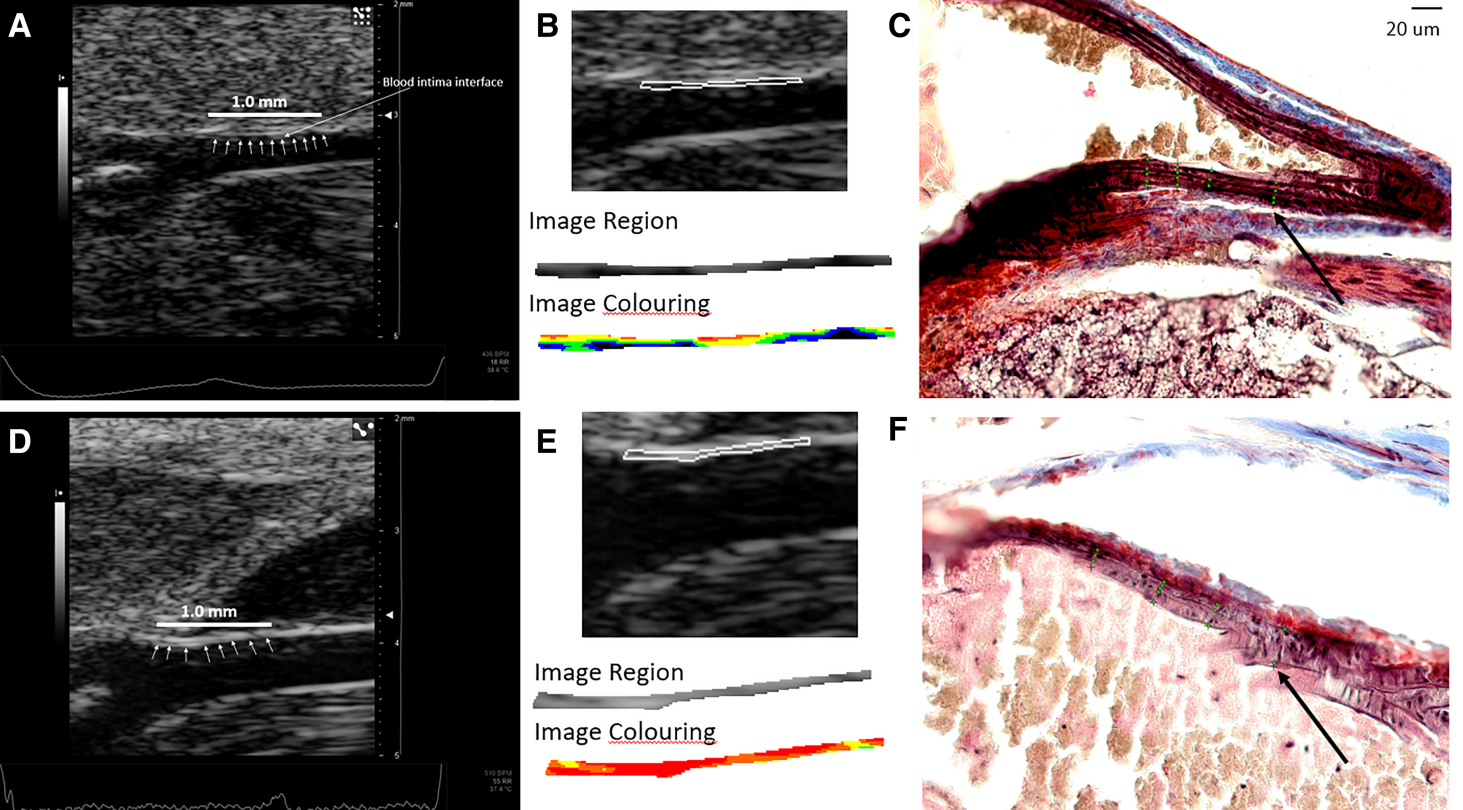
(**A–C**) Images from a male mouse at 6 weeks of age. (**A**) The normalized ultrasound image demonstrating the blood intima interface (small white arrows) for the distal 1.0 mm of the common carotid artery where the segmentation was made. (**B**) The segmented carotid artery wall and color-coded grayscale pixels for a range of grayscale values from the software (black = 0–25, blue = 26–50, green = 51–75, yellow = 76–100, orange = 101–125, red = 126–255). (**C**) The histology image with annotated elastin fiber positions (green dots along the lines perpendicular to the wall) used for measuring the number and spacing of elastin fibers. Grayscale texture feature values: GSM = 57.5, entropy = 4.49, GLDM-CON = 249.62, SGLD-ASM = 0.0014, and SGLD-HOM = 0.13. The number of elastin fibers shown in this single image is 4.26 weighted by region of interest length (arrow denotes green dots identifying elastin fibers). (**D–F**) Images from a 24-week-old male mouse. (**D**) Normalized ultrasound image demonstrating the blood intima interface (small white arrows) for the distal 1.0 mm of the common carotid artery where the segmentation was made. (**E**) The segmented carotid artery wall and color-coded grayscale pixels for a range of grayscale values from the software (black = 0–25, blue = 26–50, green = 51–75, yellow = 76–100, orange = 101–125, red = 126–255). (**F**) The histology image with annotated elastin fiber positions (arrow denotes green dots along the lines perpendicular to the wall) used for measuring the number and spacing of elastin fibers. Grayscale texture feature values: GSM = 130, entropy = 4.40, GLDM-CON = 70.16, SGLD-ASM = 0.0015, and SGLD-HOM = 0.17. The number of elastin fibers shown in this single image is 3.25 weighted by region of interest length. The black cross markers indicate the edge points of the intima–media layer. Images in panels **C** and **F** are shown at the same scale. The scale bar in **C** equals 20 µm.

**Figure 6:**
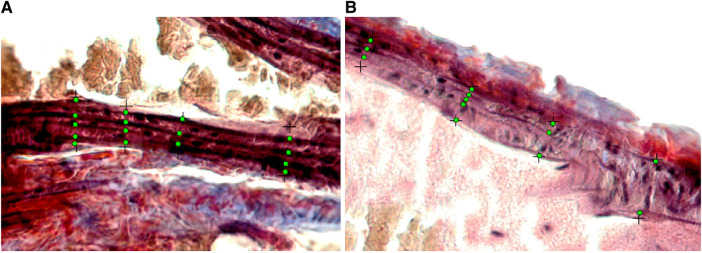
Cropped and enlarged histology images (from Figures 5C,F) showing annotated elastin fiber position (green dots). (**A**) The histology image from a male mouse at 6 weeks. (**B**) The histology image from a male mouse at 24 weeks.

**Figure 7:**
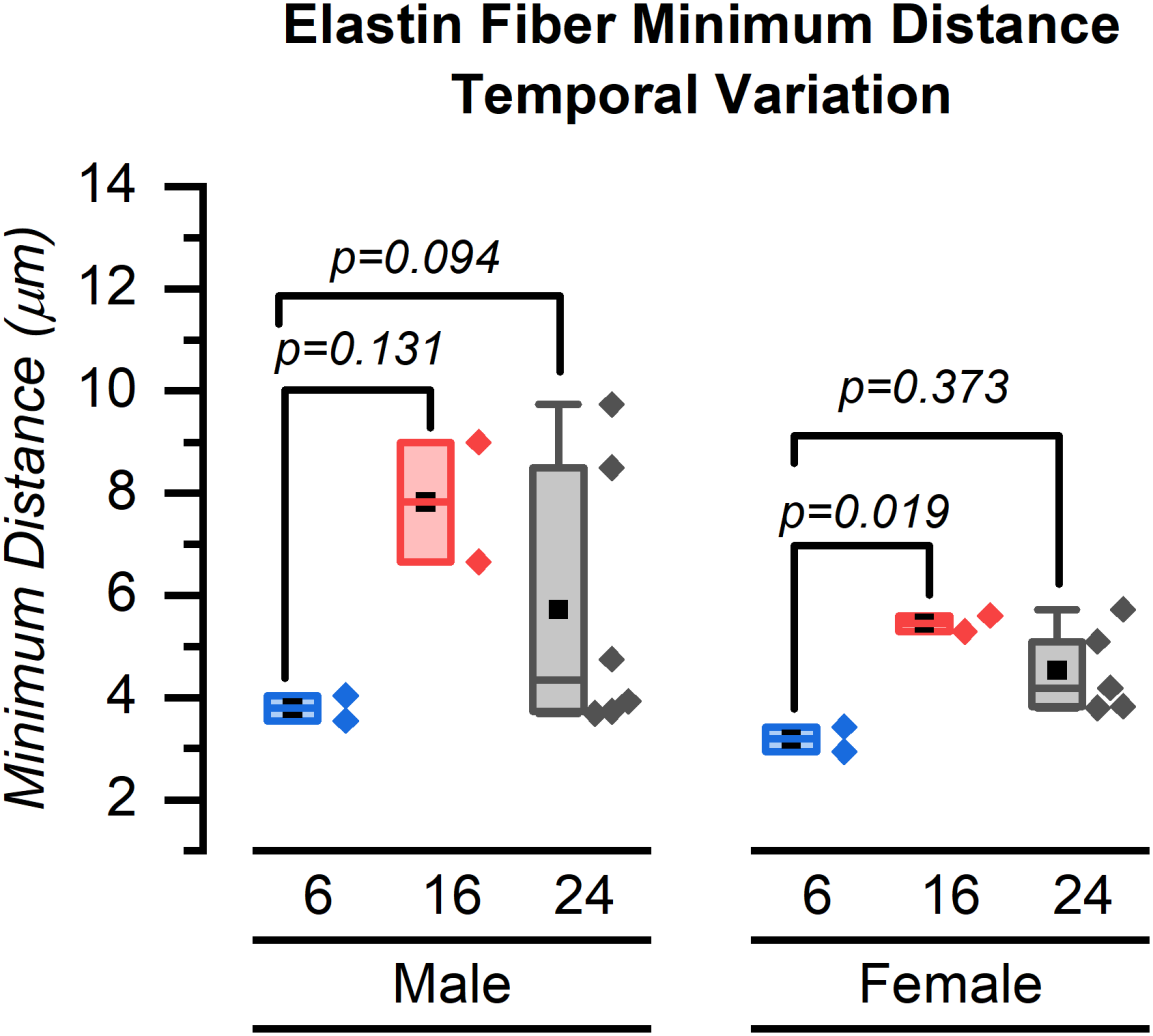
Minimum distance between elastin fibers for male and female mice at 6, 16, and 24 weeks. Symbol definitions: ▪ = mean.

Our major finding was that the number of elastin fibers in the ROI decreased from 6 weeks to 24 weeks for both male and female mice (Figures 4–6). The mean (SD) number of elastin fibers for females was 3.98 (0.38) at 6 weeks, 3.36 (0.44) at 16 weeks, and 3.46 (0.19) at 24 weeks (*p* = 0.071 and *p* = 0.051 for 6–16 and 6–24 weeks, respectively). For male mice, the mean number of elastin fibers was 5.32 (1.50) at 6 weeks, 3.08 (0.23) at 16 weeks, and 3.59 (0.38) at 24 weeks (*p* = 0.011 and *p* = 0.023 for 6–16 and 6–24 weeks, respectively) (Figure 4). Qualitatively, the male mice demonstrated a greater change in the number of elastin fibers from 6 to 24 weeks compared to that of female mice (33% vs. 13%). In addition, female mice demonstrated a significant increase in the minimum distance between elastin fibers between 6 and 16 weeks (minimum distance between elastin fibers was 3.19 (0.34) μm at 6 weeks and 5.45 (0.23) μm at 16 weeks, *p* = 0.019) (Figure 7). From 16 weeks to 24 weeks, the minimum distance between elastin fibers changed from 5.45 (0.23) to 4.53 (0.85) μm; however, this change was not significant, *p* = 0.174.

### 3.4. Sex differences in findings

For male mice, the only grayscale texture feature significantly correlated with plaque score was SGLD-HOM (*r_s_* = 0.707, *p* = 0.033) (Table 3). No other grayscale texture features significantly correlated with the number of elastin fibers or plaque score (*p* > 0.05). None of the grayscale texture features were significantly correlated with plaque score or the number of elastin fibers (all *p* > 0.05) in female mice (Table 3).

**Table 3:** Spearman rho (*r_s_*) correlation coefficient by sex between grayscale texture features, the number of elastin fibers, and plaque score.

Grayscale texture feature	Minimum distance between elastin fibers, *r*_s_	Minimum distance between elastin fibers, *p*-value	Number of elastin fibers *r_s_*	Number of elastin fibers *p*-value	Plaque score, *r_s_*	Plaque score *p*-value
Male						
Grayscale Median value	−0.250	0.516	0.000	1.00	0.228	0.555
Entropy	−0.633	0.067	0.600	0.088	−0.434	0.244
GLDM-CON	0.167	0.668	−0.150	0.700	−0.479	0.192
SGLD-ASM	−0.076	0.846	−0.279	.468	0.324	0.396
SGLD-HOM	−0.050	0.898	−0.133	0.732	**0**.**707**	**0**.**033**
Female						
Grayscale median value	−0.383	0.308	0.067	0.865	0.491	0.150
Entropy	0.350	0.356	−0.483	0.187	0.573	0.084
GLDM-CON	0.117	0.765	−0.250	0.516	0.201	0.577
SGLD-ASM	−0.035	0.929	−0.166	0.669	0.503	0.138
SGLD-HOM	−0.533	0.139	0.400	0.286	0.101	0.782

Bolded values are significant with a *p*-value < 0.05.

### 3.5. Elastin fibers and plaque score and sex

The number of elastin fibers was not significantly associated with plaque score (males *r_s_* = −0.390, *p* = 0.266; females *r_s_*_ _= −0.422, *p* = 0.258) nor were any other elastin parameters (all *p* > 0.050) (Table 4).

**Table 4:** Elastin parameters and plaque score.

Elastin measure	Plaque score (*r_s_*)	Plaque score *p*-value
Male		
Number of elastin fibers	−0.390	0.266
Minimum distance	0.202	0.575
Maximum distance	−0.127	0.726
Mean distance	−0.045	0.902
Standard deviation	−0.075	0.837
Median	−0.045	0.902
Female		
Number of elastin fibers	−0.422	0.258
Minimum distance	0.316	0.407
Maximum distance	−0.026	0.946
Mean distance	0.184	0.635
Standard deviation	0.026	0.946
Median	0.158	0.685

One male mouse at 6 weeks did not have left carotid artery measurable grayscale texture features, and one female mouse at 24 weeks did not have right-side elastin data.

### 3.6. Ultrasound reader reproducibility

One reader measured 20 mouse carotid arterial wall images twice, blinded to the first reading. Images were selected randomly at each time point to ensure that images were selected over the course of the study, and both the right and left CCAs for that time point were measured. A total of 10 images from male mice and 10 images from female mice were intentionally selected for the reproducibility study to ensure equal representation of each sex. The average time between readings was 837 (SD 49) days. The same procedure was followed at time 2 as described in the “Materials and methods” section for grayscale texture feature extraction. Images were normalized (as described above), and the pixel density was confirmed to be 190 pixels/mm. The average (SD) GSM reading for time 1 and time 2 measurements were 88.50 (30.40) units and 91.97 (29.25) units, respectively. The intra-observer absolute difference was 5.80 (4.76) units, and the intra-observer within-subject standard deviation was 5.25. The GSM intraclass correlation coefficient (ICC) was 0.97 (95% CI, 0.92–0.99). The average (SD) entropy reading for time 1 and time 2 measurements was 4.45 (0.18) units and 4.49 (0.17) units, respectively. The intra-observer absolute difference for entropy was 0.07 (0.05) units and the intra-observer within-subject standard deviation was 0.06. The ICC for entropy was 0.87 (95% CI, 0.69–0.95). The average (SD) GLDM-CON measurement for time 1 and time 2 measurements was 151.01 (68.32) and 149.16 (66.21), respectively. The intra-observer absolute difference for GLDM-CON was 14.07 (9.61), and the within-subject SD was 11.95. The ICC for GLDM-CON was 0.97 (95% CI, 0.92–0.99). The average (SD) for SGLD-ASM time 1 and time 2 measurements was 0.0015 (0.0004) and 0.0015 (0.0004), respectively. The intra-observer absolute difference for SGLD-ASM was 0.0001 (0.0001), and the within-subject SD was 0.0001. The ICC for SGLD-ASM was 0.92 (95% CI, 0.82–0.097). The time 1 and time 2 measurements for SGLD-HOM were 0.14 (0.04) and 0.14 (0.04), respectively. The intra-observer absolute difference for SGLD-HOM was 0.01 (0.01), and the within-subject SD was 0.01. The SGLD-HOM ICC was 0.95 (95% CI, 0.88–0.98).

## 4. Discussion

As novel grayscale ultrasound image analysis techniques become increasingly used in research studies, it is important to understand what these measures represent at a tissue level and how their changes, which reflect changes in CVD risk, reflect changes at the tissue level. In this study, we used a murine model that is designed to develop atherosclerosis (22). Our main finding was that elastin fibers decreased with age in both male and female mice, although this finding only was significant in male mice. We also demonstrated that changes in the ultrasound grayscale texture feature SGLD-HOM were associated with the plaque score.

Male mice demonstrated a significant decrease in elastin fibers from 6 to 24 weeks. Arterial wall homogeneity for the male mice also was associated positively with plaque score, indicating that arteries that are more homogenous are associated with greater atherosclerotic plaque burden. Female mice showed a significant decrease in GSM from baseline 6 weeks to 16 weeks and an increase, although not significant, in GSM from 6 weeks to 24 weeks yet also developed plaque and had plaque scores higher than those of male mice at 24 weeks. Other pre-clinical mouse studies examining plaque formation in the aortic root and aorta have demonstrated that female mice have larger plaque areas compared to those of male mice that were fed atherogenic diets and sacrificed between 24 and 26 weeks of age (34–38). Our mice were euthanized at 24 weeks, and thus, this finding of higher plaque scores in our female mice at 24 weeks is similar to what others have reported regarding differences between plaque area and sex and provides external validity to our model and novel observations about the grayscale measures.

In both human (39) and pre-clinical animal studies (40, 41), males develop plaques with increased inflammatory cell content compared to females (34, 39–41). Thus, increased homogeneity in our mice may indicate early inflammatory changes associated with early atherogenesis changes prior to plaque presence and/or with plaque present in the carotid arteries of male mice and may explain why the texture changes in this study were different in male mice compared to female mice. Our ongoing experiments are examining how lipid content and inflammatory cell infiltrates are associated with grayscale features in our mouse models.

As mice age, elastin degrades (42). In our study of Apoe^tm1Unc^ mice, we identified a quantifiable change in elastin fiber number from 6 to 16 and 24 weeks, which is much earlier than reported by others (42). However, we used a murine model that is designed to develop atherosclerosis (22). At week 6, there were more elastin fibers present, and histological analysis of the artery demonstrated a pattern of smooth muscle with relatively regularly interspersed elastin. With elastin fragmentation, there was evidence of wider spacing between elastin fibers, and this results in a structure in which the tissue is more homogenous (i.e., a greater percentage of smooth muscle cells relative to elastin fibers). This may result in an arterial wall with increased homogeneity due to the tissue being more similar, thus resulting in tissue that has similar grayscale values in the pixels comprising an ultrasound image.

We did not see a significant association between plaque score and elastin fiber number. This could be due to the fact that we were quantifying the elastin number in the plaque-free segment of the arterial wall and not at the level of the actual plaque. This was done purposefully in this study to examine how the structure of the arterial wall (i.e., elastin fiber number and spacing) changed from baseline to 24 weeks in the mice and to see if these changes were associated with grayscale texture features. Others (43, 44) reported that impaired elastin structure of the vessel wall was associated with the progression of atherosclerosis in ApoE^−/−^ C1039G^+/−^ crossbred mice compared to ApoE^−/−^ mice (43, 44). However, this group evaluated plaque at the level of the aortic root, brachiocephalic artery, and descending aorta with histology results in a mouse model that was bred to have a mutation in the fibrillin-1 gene (44). Mutations in this gene are associated with elastic fiber fragmentation (44). This group further demonstrated that fragmentation of elastic fibers was associated with increased vascular stiffness and features of vulnerable plaque (43, 44). The results of this study are promising and support what has been demonstrated in human studies where arteries that have lower contrast and are more homogenous are associated with abnormal arterial findings that may indicate an increased risk for adverse CVD events (1). These results also support differences seen in pre-clinical studies based on sex (34, 35, 39–41). Both our and others’ (1–4) findings suggest that a more worrisome arterial phenotype may be one in which the GSM, entropy, and contrast are low and the measure of homogeneity is high. We believe that our findings may serve as a foundation for how texture can be used to describe arterial phenotypes associated with early structural changes and atherogenesis and to examine how these changes differ by sex.

## 5. Limitations

The number of mice we studied was small; however, our preliminary findings identified several significant associations between grayscale measures, plaques, and elastin, indicating a consistent effect size for several measures. It is likely that even more associations would have been statistically significant with a larger sample size, since several of the correlations had a moderate effect size of >0.30, but the corresponding *p*-values were not statistically significant. Because we used atherosclerosis-prone Apoe^tm1Unc/J^ mice that were fed a high-fat diet to intentionally accelerate atherogenesis, we cannot separate changes in elastin fibers associated with aging from those associated with atherogenesis in this mouse model. We only studied mice that were prone to atherosclerosis since we were studying arterial injury and studying arteries that were healthy over time was not a prudent use of animal resources given the scientific questions to be answered.

## 6. Conclusion

Grayscale ultrasound texture features that are associated with increased risk for CVD events in humans were used in a murine model, and the grayscale texture feature SGLD-HOM was shown to change in male mice from 6 to 24 weeks. Structural alterations of the arterial wall (change in elastin fiber number) that reflect arterial injury during atherogenesis also were observed during this time frame and may differ by sex.

## Data Availability

The original contributions presented in the study are included in the article/Supplementary Material, further inquiries can be directed to the corresponding author.
